# Plant responses to climate change, how global warming may impact on food security: a critical review

**DOI:** 10.3389/fpls.2023.1297569

**Published:** 2024-01-05

**Authors:** Michela Janni, Elena Maestri, Mariolina Gullì, Marta Marmiroli, Nelson Marmiroli

**Affiliations:** ^1^ Institute of Bioscience and Bioresources (IBBR), National Research Council (CNR), Bari, Italy; ^2^ Institute of Materials for Electronics and Magnetism (IMEM), National Research Council (CNR), Parma, Italy; ^3^ Department of Chemistry, Life Sciences and Environmental Sustainability, Interdepartmental Centers SITEIA.PARMA and CIDEA, University of Parma, Parma, Italy; ^4^ Consorzio Interuniversitario Nazionale per le Scienze Ambientali (CINSA) Interuniversity Consortium for Environmental Sciences, Parma/Venice, Italy

**Keywords:** global change, food security, global warming, holistic approach, omics, sustainability

## Abstract

Global agricultural production must double by 2050 to meet the demands of an increasing world human population but this challenge is further exacerbated by climate change. Environmental stress, heat, and drought are key drivers in food security and strongly impacts on crop productivity. Moreover, global warming is threatening the survival of many species including those which we rely on for food production, forcing migration of cultivation areas with further impoverishing of the environment and of the genetic variability of crop species with fall out effects on food security. This review considers the relationship of climatic changes and their bearing on sustainability of natural and agricultural ecosystems, as well as the role of omics-technologies, genomics, proteomics, metabolomics, phenomics and ionomics. The use of resource saving technologies such as precision agriculture and new fertilization technologies are discussed with a focus on their use in breeding plants with higher tolerance and adaptability and as mitigation tools for global warming and climate changes. Nevertheless, plants are exposed to multiple stresses. This study lays the basis for the proposition of a novel research paradigm which is referred to a holistic approach and that went beyond the exclusive concept of crop yield, but that included sustainability, socio-economic impacts of production, commercialization, and agroecosystem management.

## Global warming, temperature stress and eco-physiological effects on crop yield and quality

Climate change and agricultural production are highly correlated. It is now well established that global warming affects agriculture in several ways, including changes in average temperatures and rainfall. The predictability of extreme meteorological events (e.g. heat waves, flood and drought), changes in pests and diseases, increase in atmospheric carbon dioxide and ground-level ozone concentrations, and changes in the nutritional quality of foods (Zhao et al., 2020; Kumar et al., 2022) are among the drawbacks of this phenomena.

This study considers the relationship of climatic changes and their bearing on sustainability of natural and agricultural ecosystems, with a consideration to the role of omics-technologies, genomics, proteomics, metabolomics, phenomics and ionomics. Improving crops for higher adaptability and tolerance to climate changes can be achieved by resource saving technologies as precision agriculture and new fertilizers and amendments. Nevertheless, the adoption of a more holistic vision of agriculture and food production is necessary to achieve sustainable food security.

Global warming is defined as the continuing rise of the average temperature of the Earth’s climate system and is one of the cause forcing climate change (IPCC, 2019; Seneviratne et al., 2021; Zandalinas et al., 2021). Temperature is one of the major environmental factors affecting plant growth, development, and yield. Temperatures persistently above those optimal for plant growth may induce heat stress (HS), thus constraining the flowering and fruit developmental processes and strongly reducing yields. At some threshold high temperature may cause plant death. Extreme heat events can be classified according to the maximum temperatures reached (intensity), how often the events occur (frequency), and how long they last (duration). Extreme HS episodes and prolonged heat (global warming) demand radically different approaches from breeders to meet the demands of farmers, and consumers for food security. Several aspects need to be considered when carrying out risk assessment for crop production and food security. These include the extent of the adverse event, how frequently the sustainable temperature thresholds are likely to be crossed within the growing season, whether these extreme episodes exceed lethal temperatures, and the length of the event. Models, that capture the variety of drivers determining crop yield variability and scenario climate input data that samples the range of probable climate variation have been developed with an eye towards the mitigation of yield losses (Ribeiro et al., 2020; Schauberger et al., 2021; Stella et al., 2021). Under a global warming scenario, the identification of the temperature thresholds for the major crop plants and their effects on yield is vital in predicting risk for food security (Zhao et al., 2017).

This is particularly true when considering that frequency and intensity of heat events will increase dramatically in the future, especially in tropical regions (geographic perspective) and in developing countries (national perspective) leading to >15% of global land becoming more exposed to levels of heat stress that will affect both food production and human health (Sun et al., 2019).

**Figure 1:**
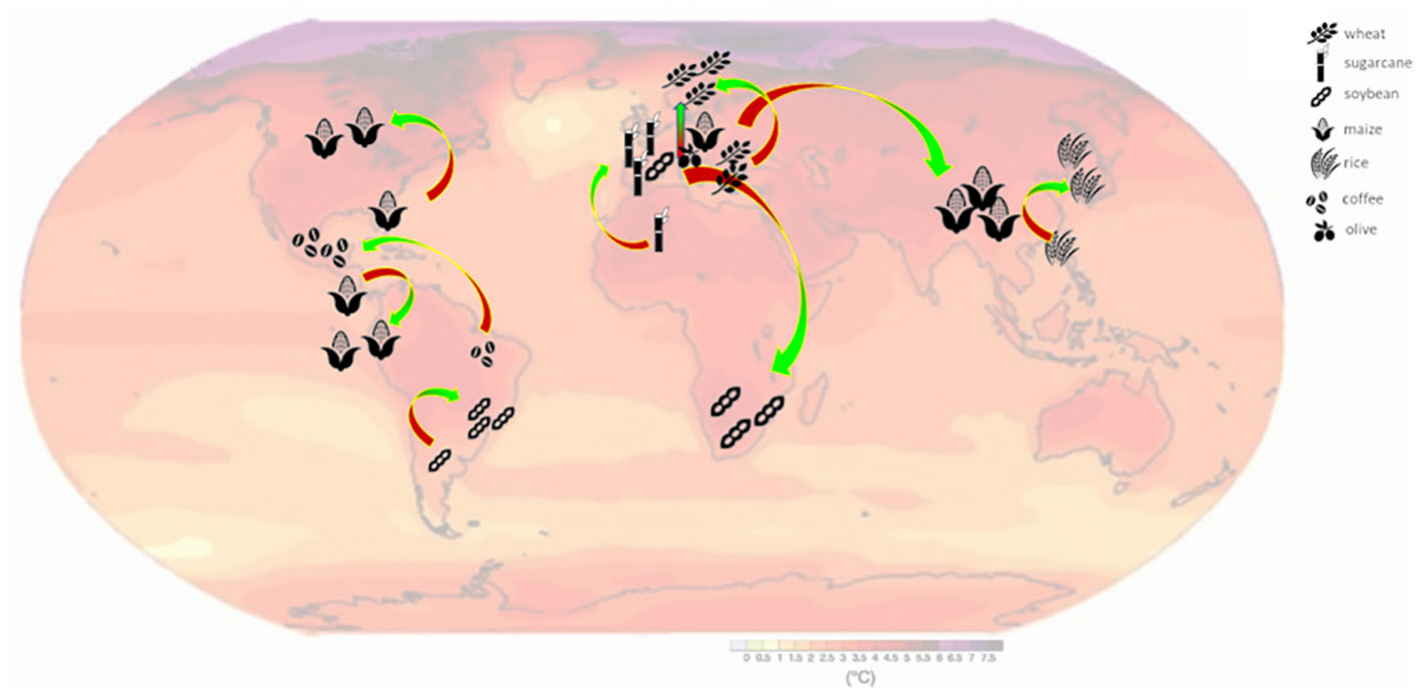
Map of probable shifts in cultivation areas of some key and traditional crops due to global warming and climate change, positioned on the heat prevision map extracted from the last IPPN report (2081-2100).

Food production in the last century has shifted from the use of about 2500 different plant species to reliance on the ‘four queens’: rice, wheat, maize, and soybean (Smýkal et al., 2018) (**Figure 1**). These crops provide two-thirds of the total human energy intake, while the grain legumes alone contribute 33% of required human dietary proteins. This affects food security and environmental sustainability (Foyer et al., 2016). Persistent dependence on such a small number of agricultural commodities (Khoury et al., 2014) coupled with climate uncertainties (Foley et al., 2011) could become factors of great economic instability and political vulnerability. Assessing the impact of global temperature increases on the production of these commodity crops is therefore a critical step for maintaining the global food security (Zhao et al., 2017) as discussed in recent reviews reporting on the threshold temperatures for several crop species (Kaushal et al., 2016; Janni et al., 2020).

Several examples have been reported of the effects of heat on crop yield and quality. In wheat a mean daily temperature of 35°C caused total failure of the plant, while exposure to short episodes (2–5 days) of HS (>24°C) at the reproductive stage (start of flowering) resulted in substantial damage to floret fertility leading to an estimated 6.0 ± 2.9% loss in global yield with each degree-Celsius (°C) increase in temperature [8, 35]. Increasing the duration of high temperature at this stage linearly reduced the grain weight (Prasad and Djanaguiraman, 2014); similarly for pea (Bhattacharya, 2019), lentil (Barghi et al., 2012) and chickpea (Wang et al., 2006). In response to 2°C of global warming, the total production in the top four maize-exporting countries is projected to decline by 53 million tons (51.9–54.8), equivalent to 43% (41.5–43.8) of global maize export volume (Tigchelaar et al., 2018). Kaushal et al. (2016) (Kaushal et al., 2016) provide an extensive analysis for several crop species of the threshold temperatures above which growth and development are compromised, while Zhou et al. (2022) (Zhou et al., 2022), extensively reported the physiological effects of heat stress on yield limitation (Zhou et al., 2022). A recent overview of the effects of threshold temperatures for vegetative growth and reproductive development in several crop species has been reported by Janni and co-workers (2020) (Janni et al., 2020). Even taking into account the heterogeneity in the collection of data and the time frames of experiments, it is evident that HS is correlated with decreased yields of the major crops; cereals are particularly sensitive to heat during grain filling, which also affects the quality (Maestri et al., 2002). Seed filling is a crucial growth stage for most crops, and involves mobilization and transport of various chemical constituents, and activates many biochemical processes made for the synthesis of proteins, carbohydrates, and lipids in the developing seeds (Ali et al., 2017). It is influenced by various metabolic processes occurring in the leaves, especially production and translocation of photo-assimilates, providing precursors for biosynthesis of seed reserves, minerals, and other functional constituents (Fahad et al., 2017; Sehgal et al., 2017).

HS can impair several physiological processes linked with seed size and quality. HS during grain filling markedly decreases starch accumulation (Hurkman et al., 2003), in rice (Yamakawa and Hakata, 2010) and maize (Yang et al., 2018) as well as the levels of sugars such as fructose and sugar nucleotides as hexose phosphate (Yang et al., 2018); the decrease in sugars may be related to enhanced assimilate utilization rather than to an increase in edible component production. In maize, waxy grain starch content was decreased, whereas protein content was increased, resulting in a change of grain quality (Yang et al., 2018). Moreover, increasing temperature and CO_2_ induces protein and micronutrients contents in grain (Chakraborty and Newton, 2011) and in soybean (Li et al., 2018). In soybean under HS, total free amino acids were reduced together with the total protein concentration, while the oil concentration was significantly increased (Takahashi et al., 2003). As a general conclusion, under HS, reductions in total yield are mainly due to alteration of the source and sink activities that take place.

Although it might be argued that the ‘fertilization effect’ of increasing CO_2_ concentration may benefit crop biomass thus raising the possibility of an increased food production (Degener, 2015), emerging evidence has demonstrated a reduction in crop yield if increased CO_2_ is combined with high temperature and/or water scarcity, making a net increase in crop productivity unlikely (Long et al., 2006). Water supply is thus a deeply linked issue. It has been estimated that in the period 1990-2020 total rainfed and irrigated growing areas together increased by 35% for maize, 0.3% for wheat, 13% for rice, and 159% for soybean. Rainfed areas for wheat and rice decreased by 10 and 7%, respectively, while the rainfed maize area increased by 24% (compared to the 35% increase in total area), and rainfed soybean areas increased by 158% - most of the increase in soybean areas was rainfed (Sloat et al., 2020).

Moreover, each 1°C rise in the global mean temperature reduces global maize yield by 7.4%, wheat yield by 6.0%, rice yield by 6.2%, overall milled rice by 7.1–8.0%, head rice by 9.0–13.8% and overall milling profit by 8.1–11.0% and soybean yield by 3.1% (Parthasarathi et al., 2022).

When the combination of drought and heatwave is considered, production losses considering cereals including wheat (−11.3%), barley (−12.1%) and maize (−12.5%), and for non-cereals: oil crops (−8.4%), olives (−6.2%), vegetables (−3.5%), roots and tubers (−4.5%), sugar beet (−8.8%), among others (Brás et al., 2021).

## Agroecosystems resilience, plant resilience, temperature tolerance

An increases of global temperature was perceived already in the 70s and lead to the definition of this phenomena as global warming (Broecker, 1975). Indeed, the majority of reports have warned that HS due to increases in global temperature can cause global yield to a decline (Sadok and Jagadish, 2020; Zhu et al., 2022) as a result of eco-physiological stress.

In fact, projections of climate change risks produced through advanced modelling are consistent in indicating a negative influence on crop production (Challinor et al., 2014; Konduri et al., 2020) and a worsening in food quality and nutritional values (Chakraborty and Newton, 2011). Climate models can forecast temperature increases at the regional level with higher certainty than other changes, as precipitation. Multimethod analysis can improve our confidence in assessment of some aspects and consequences of future climatic impacts on crop productivity and inform about the adoption of specific rescue strategies (Zhao et al., 2017). After 30 years of efforts and some progress under the United Nations Framework Convention on Climate Change (UNFCCC), the anthropogenic greenhouse gas (GHG) emissions in continue increases and the evenience of a catastrophic exit is relatively under-studied and poorly understood (Kemp et al., 2022).

The specialization in crop selection and production, and the economic scale that has developed, have led to a huge increase in productivity in agroecosystems. But the long-term sustainability of these may be reduced by some of the constraints associated with global warming, especially when it is considered what the current complex agroecosystems provide not only for harvest, but also for other important ecosystem services of great social and economic value (Di Falco and Chavas, 2008).

Several reviews have addressed mainly HS effects on crop yield, focusing on the role played by the molecular mechanisms underpinning plant resilience and yield reduction (**Table 1**). However, most did not consider global warming and HS as significant combinatorial factors (**Table 1**) in acting to reduce food security.

**Table 1:** Recent reviews and articles focused mainly on heat stress and effects on crop yield and the main components of defense responses.

Author	Type of article	Focus	Reference #
Pareek et al., 2020	Special issue	Mitigating the impact of climate change on plant productivity and ecosystem sustainability.	(Pareek et al., 2020)
Lohani et al., 2020	Review	Molecular mechanisms that contribute to temperature sensitivity are ably discussed and a summary presented of the regulation of male and female reproductive organ development and fertilization, together with heat-induced abnormalities at flowering.	(Lohani et al., 2020)
Sharma et al., 2020	Research article	Importance of plant growth regulators (PGRs) as protection against high-temperature stress (HTS)	(Sharma et al., 2020)
Janni et al., 2020	Review	Molecular and genetic bases of heat stress responses in crop plants and breeding for increased resilience and productivity.	(Janni et al., 2020)
Venios et al., 2020	Review	Heat stress and global warming impacts on grapes.	(Venios et al., 2020)
Lima et al., 2021	Review	Effects on heat on agricultural workers’ health.	(de Lima et al., 2021)
Malhi et al., 2021	Review	Climate change effects and projections in the near future, together with their impact on the agriculture sector as an influence on physiological and metabolic activities of plants. Implications for growth and plant productivity, pest infestation, and mitigation strategies and their economic impact.	(Malhi et al., 2021)
Zandalinas et al., 2021	Review	Impact of a multifactorial stress combination on plants, soil, and microbial populations.	(Zandalinas et al., 2021)
Brás et al., 2021	Review	The severity of drought and heatwave crop losses has tripled over the last five decades in Europe. The review gives an overall picture of the progression of the climate disaster and its impact on crop yield.	(Brás et al., 2021)
Ul Hassan et al., 2021	Review	summarizes the alterations in the development systems of plants in response to heat stress with a focus on integrated morpho-anatomical, physiological, and molecular adaptations. It also provides information about advanced heat tolerance mechanisms in various plant species applying different tactics together with genetic techniques for plant growth and development	(Ul Hassan et al., 2021)
Yadav et al., 2022	Review	Impacts, Tolerance, Adaptation, and Mitigation of Heat Stress on Wheat under Changing Climates	(Yadav et al., 2022)
Zhao et al., 2022	Review	The study highlights the importance of modeling crop yields under heat stress to food security, agricultural adaptation, and mitigation to climate change.	(Sun et al., 2022)
Han et al., 2022	Review	The review reports the literature related to response and tolerance mechanism of food crops	(Han et al., 2022)
Zhou et al., 2022	Review	The review reports the current study of crops at abiotic stresses in particular heat stress using omics	(Zhou et al., 2022)
Zhou et al., 2022	Review	Heat-responsive molecular regulatory pathways mediated, respectively, by the Heat Shock Transcription Factor (HSF)–Heat Shock Protein (HSP) pathway and PHYTOCHROME INTER-ACTING FACTOR 4 (PIF4) pathways	(Zhou et al., 2022)
Saeed et al., 2023	Review	The review reported the effects of heat stress on vegetables and highlights recent research with a focus on how omics and genome editing	(Saeed et al., 2023)

Few reviews tackle global warming and climate change’s effects on agriculture.

Resilience of cropping systems to global warming and to temperature increase can be described in terms of resilience of the related agroecosystems, i.e. their capacity to support yield in critical environmental conditions like HS (Allan et al., 2013; Zampieri et al., 2020; Saeed et al., 2023). We can think the resilience of an ecosystem as the capacity to maintain its function, identity and organization, though subjected to a critical disturbance (Holling, 1978). For agroecosystems this definition is problematic due to the bias of human intervention, but metrics of resilience can be taken into consideration in a framework which uses a number of phenological indicators (Cabell and Oelofse, 2012; Deutsch et al., 2018).

Resilience is certainly a holistic way to describe some properties of agroecosystems which are context-dependent (Carpenter et al., 2001). But a system considered resilient today can become less so over the years or even the months, because of a gradual or a sudden changes of context (Holling, 2001). Tolerance to temperature stress has a cost because it implies a consistent allocation of energy resources to maintain survival at the expense of reproduction and growth and therefore with a tradeoff between maintenance and yield.

Three mutually interacting concepts need to be considered when dealing with agroecosystems. These are (i) agroecosystem welfare and the way it interacts with human needs over the time; (ii) agroecosystem resilience, meaning its capacity to adapt, overcome stress and reorganize in stressing environments or when perturbation to the norm becomes frequent, as in global warming; and (iii) food security, the production of sufficient food of good quality for the human and animal populations. A holistic approach to food security expands the problem well beyond the simple concept of crop yield, also including sustainability, socio-economic impacts of production, commercialization, and agroecosystem management.

Both social and biological aspects are relevant to a correct management of agroecosystems. But climate change and global warming could give rise to such a rapid, deep, and unpredictable changes that current agroecosystems may fail to adapt. Recently, a meta-analysis on 10,000 animal species has been published considering only phenological traits, concluding that most of these species are at a risk of not surviving if global change continues in intensity and direction. Even maintaining the highest possible level of diversity within our agroecosystems may not be sufficient to combat global change and its effects on food security (Hoy, 2015).

Global warming and temperature increase are often taken as stressor examples but although they are certainly threatening phenomena, it is difficult to isolate each single component from them. Plants resilient to global warming and temperature increase may be capable of withstanding HS without any significant departure from their growth habits and productivity (Maestri et al., 2002; Law et al., 2018).

## Novel fertilizers and biostimulants to increase plant resilience

As previously widely discussed, global changes including high temperatures, drought, and salt accumulation are reported main factors of soil desertification and plants yield reduction. In this context, biostimulants (BSts) could play crucial roles in mitigating the negative effects of stresses on plants by inducing several protection mechanisms, like molecular alteration and physiological, biochemical, and anatomical modulations (Sangiorgio et al., 2020; Bhupenchandra et al., 2022). They also stimulate the innate immune responses of plants to biotic stress by deploying cellular hypersensitivity, callose deposition, and lignin synthesis (Bhupenchandra et al., 2022).

Production of “conventional” chemical fertilizers has a large share in global CO2 emission, calculated in about 500 million tons/year (FAO, 2020) worldwide. Production of organic fertilizers on the other hand is largely dependent on animal farming with its considerable share of glass house gas emission (Timsina, 2018; Ramakrishnan et al., 2021) Sustainable alternatives under experimentation are nanofertilizers (Kah et al., 2018), biofertilizers (Bhardwaj et al., 2014) and new soil amendments (Rombel et al., 2022).

Nanofertilizers belong to the family of engineered nanoparticles (ENPs) with dimension between1-100nm and have shown some beneficial protecting effects on plants, like stimulation of growth and promotion of nutrients absorption (Abdel-Aziz et al., 2021; Kalwani et al., 2022). Recent studies on tomato have shown the beneficial effect of some nanoclay which confirmed previous studies in zucchini (Marmiroli et al., 2021; Pavlicevic et al., 2022).

Some advantage of nanofertilizers as compared to chemical fertilizers are the slower release of the nutrients with the time thus avoiding dispersion and washing out to superficial water body with risk of eutrofization (Zulfiqar et al., 2019). However, the production of nanofertilizers is still expensive and limited by regulatory frameworks and then use by farmer’s acceptance (Kah et al., 2018; Kah et al., 2019). Biological (Green) synthesis of Bio Nanofertilizers is very slow but may became a suitable option (Zulfiqar et al., 2019).

Plant growth promoting microorganisms (PGPM) are types of microbes (bacteria, fungi) that through a plant-microbe interaction stimulates the plant immune system (Backer et al., 2018). PGPM stimulates and enhances plant capabilities to absorb nutrients and defend from pathogens. This may result in increased plant yield and health (Backer et al., 2018; Lopes et al., 2021; Ramakrishnan et al., 2021). The performance of biofertilizers can be enhanced by combining them with soil amendments that have the positive characters of improving soil properties (pH, CE, water holding capacity) and stimulate microbial growth (Backer et al., 2018; Mohamed et al., 2019; Rouphael and Colla, 2020).

Among the newly developed soil improvers, biochar has gained same interest because: i) it is produced by pyrolysis or pyrogasification of removable biomasses which does produce no significant amounts of CO2, ii) on the contrary, once in the soil, increases significantly the soil CO2 holding capacity, iii) has a high porosity and absorbent capacity toward water, nutrients and iv) can provide a reliable niche for PGPM, thus favoring their persistence and growth in the soil after inoculation. Recently it has been found in wheat and maize that biochar “functionalized” with PGPMs favor the soil microbial diversity and the cross talk between plant and soil which leads to better plant physiological parameters (Graziano et al., 2022). A matrix evaluating risks and benefits in biochar utilization has been recently proposed (Marmiroli et al., 2022).

The relevance of these new BSts for the nutrition and health of plants in the condition of global warming is paramount. They increase the natural resilience of the plant against environmental clues (biotic and abiotic) through the stimulation of the plant immune system, potentiate the water holding capacity of the soil like “pore water” (Beesley et al., 2010)and therefore expose the plant to a low water stress, determining a slower release of nutrients and making the same more broad available from the plant.

An important consideration was also for the global savings in CO2 emission, which their introduction in agriculture may determine (Li and Chan, 2022).

## Recent updates in omics for heat resilience

Many novel omic technologies, including genomics, proteomics, metabolomics, phenomics and ionomics, have been applied during the last few decades to investigate the modifications in the genome, transcriptome, proteome, and metabolome occurring as plant stress conditions change (Wani, 2019). Omic technologies provide independent information about the genes, genomes, RNAomes, proteomes and metabolomes; however, integrating these information is important for finding a durable solution to the questions addressed. A typical “integromics” study on the stress-responsive behavior of a given crop examines the genes and genome to understand their structure and organization and identifies candidate genes using either structural or functional genomics (Muthamilarasan et al., 2019), as well as data from metabolomics.

The progress of omics technologies has enabled direct and unbiased monitoring of the factors affecting crop growth and yield in response to environmental threats (Janni et al., 2020; Raza et al., 2021b; Raza et al., 2021a). Overall, omics constitute powerful tools to reveal the complex molecular mechanisms underlying plant growth and development, and their interactions with the environment, which ultimately determine yield, nutritional value (Setia and Setia, 2008; Soda et al., 2015), and the required level of agricultural inputs. Janni et al. (2020) reported an exhaustive list of success in case studies focused on the application of omics to several crops to enhance crop resilience to HS (Zhou et al., 2022).

Ionomics is a high-throughput elemental profiling approach which studies the mechanistic basis in mineral nutrient and of trace elements composition (also known as the ionome) of living organisms (Pita-Barbosa et al., 2019). By coupling genetics with high-throughput elemental profiling, ionomics has led to the identification of many genes controlling the ionome and of their importance in regulating environmental adaptation (Huang and Salt, 2016; Zhang et al., 2021).

Most genomics investigations are concentrated to understanding the role of Heat Shock Proteins (HSPs) and Heat Shock Factors (HSFs) in heat response in crops such as tomato (Scharf et al., 2012; Marko et al., 2019), in barley (Mangelsen et al., 2011) and wheat (Maestri et al., 2002; Hurkman et al., 2013; Comastri et al., 2018), with a focus on flower development and flowering time. Reactive Oxigen Species (ROS) genes also play a key role in basal heat tolerance, alone or as regulators of the activation of HSF (Driedonks et al., 2016) and therefore are considered with equal interest.

Other reviews have discussed the identification of differentially expressed genes (DEGs) associated with heat stress (Masouleh and Sassine, 2020; Wang et al., 2020; Zhao et al., 2020; Kang et al., 2022). Proteomics has provided detailed information for the encoded proteins, revealing their function in stress tolerance mechanisms (Priya et al., 2019; Katam et al., 2020) in several plant species and developmental stages (Janni et al., 2020; Chaturvedi et al., 2021). Adaptive response to HS also involves various post-translational modifications (PTMs) of proteins. The accumulation of stress-associated active proteins (SAAPs) in wheat has been reported recently (Kumar et al., 2019).

New breeding techniques (NBTs) and in particular those based on genome editing (CRISPR/Cas9) encompasses an impressive and revolutionary set of molecular tools to enhance productivity by creating genetic variability for breeding purpose, disease-free and healthy planting genetic material, improvement in stress tolerance (Mote et al., 2022; Brower-Toland et al., 2023; Liu et al., 2023). The genome-editing approach can significantly accelerate the breeding times to select environmentally tolerant crop varieties (Zhang et al., 2023).

It is now well established that major environmental stress causes metabolic reorganization towards homeostasis, maintaining essential metabolism and synthesizing metabolites with stress-protective and signaling characteristics (Schwachtje et al., 2019). This has been determined applying untargeted metabolomics in species including tomato (Paupière et al., 2017), maize (Qu et al., 2018), barley (Templer et al., 2017), wheat (Thomason et al., 2018; Buffagni et al., 2020; Yadav et al., 2022), soybean (Xu et al., 2016), citrus (Zandalinas et al., 2017) and rice (Sun et al., 2022). Sugars, free amino acids, antioxidants, fatty acids and organic compounds are key players in the heat response and in the response to combined stresses such as heat plus drought (Vu et al., 2018). Furthermore, lipids, being major components of cells and organelles membranes, are among the first targets of ROS produced during HS (Narayanan et al., 2016; Narayanan et al., 2018). An interesting correlation was found between the type of metabolites involved and the need to protect specific cellular functions or cell compartments from the adverse effects of stress, drawing attention to the application of metabolomics approaches for identification of new genetic materials for breeding.

Improvements have been achieved in recent years using plant phenomics as a tool to mitigate global warming effects and shaping genotypes and varieties more adaptable to the ongoing environmental challenges. Plant phenotyping enables non-invasive quantification of plant structure and function and interactions with their environment and can be employed in pre-breeding and breeding selection processes (Watt et al., 2020). Modern plant phenotyping measures complex traits related to growth, yield, and adaptation to stress, with an improved accuracy and precision at different scales of organization, from organs to canopies (Fiorani and Schurr, 2013). High throughput phenotyping (HTP) involves the acquisition of digital phenotypic traits by means of sensors, typically in the visible spectrum, as well in the near infrared, and in the induced fluorescence domain (Tardieu et al., 2017), to monitor plant photosynthetic activity (Li et al., 2014; Perez-Sanz et al., 2017), growth status (Petrozza et al., 2014; Danzi et al., 2019) and overall water content as main components of plants’ response to stress. HTP has been used successfully to monitor heat stress in plant species including rice, wheat and *Arabidopsis* and to select stay-green genotypes (Araus and Kefauver, 2018; Juliana et al., 2019; David et al., 2020; Gao et al., 2020; Karwa et al., 2020; Karwa et al., 2020; Luan and Vico, 2021; Pettenuzzo et al., 2022).

Successful image-based methods have been developed that directly target yield potential traits, in particular by increasing the throughput and accuracy of enumerating wheat heads in the field to help breeders manipulate the balance between yield components (plant number, head density, grains per head, grain weight) and environmental conditions in their breeding programs (David et al., 2020).

The application of biosensors in the field and under controlled environment conditions increases comprehension of the mechanisms underlying ionomics and metabolomics and can markedly improve the efficiency of water management as well as informing breeders of the most resilient genotypes (Coppedè et al., 2017; Janni et al., 2019).

The perception that inadequate phenotyping methods can hinder genetic gain in major crops has aroused the interest of the scientific community and the launch of national, regional, and international initiatives (Araus et al., 2018) such as IPPN (https://www.plant-phenotyping.org/), EPPN2020 (eppn2020.plant-phenotyping.eu) and EMPHASIS (https://emphasis.plant-phenotyping.eu/). With the increased availability of large-scale datasets, deep learning has become the state of the art approach for many computer vision tasks involving image-based plant phenotyping (Singh et al., 2018; Alom et al., 2019; David et al., 2020) allowing the development of powerful image-based models.

## A holistic thinking within knowledge-based strategies to tackle with global changes

**Figure 2:**
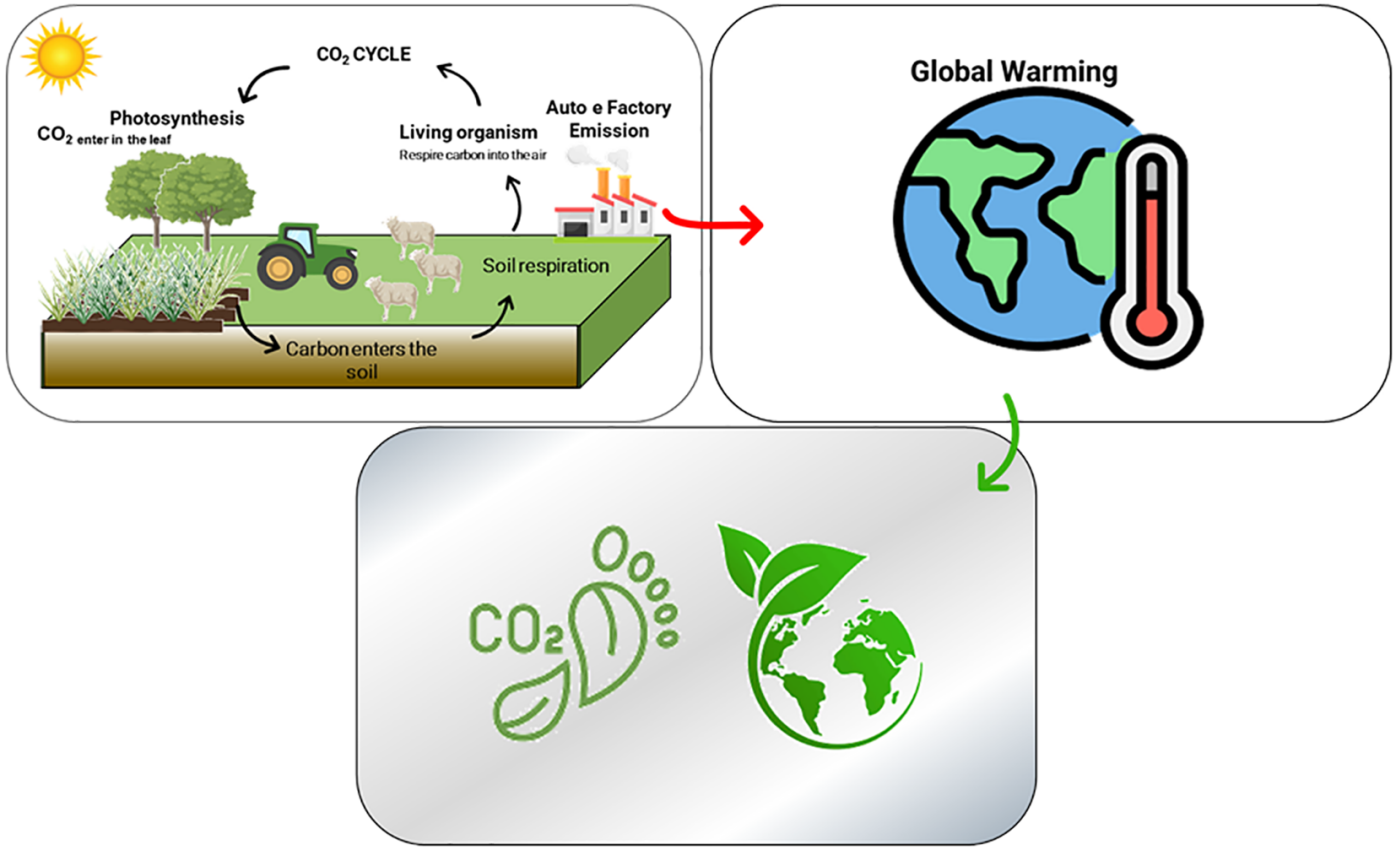
Soil-CO_2_ emissions-Global Warming and Sustainability connection with an holistic view.

Soon, temperature increases, and global warming are significantly affect the economy and all other aspects of life. Occasional heat waves have always been an aspect of summer weather in many areas of the world; but as climate change makes heat waves more frequent and more intense, the consequent risks for the agriculture sector need to be rethought strategically (**Figure 2**). The economic drawback of prolonged exposure to heat on a quantity measure of output in agriculture is stronger. Specifically, an abnormally hot day proceeded by at least eight others reduces the FAO Crop Production Index by almost 3%. Heat-wave measure implies per-wave reductions in output ranging from $0.8–3.1 billion for agriculture and up to $31.9 billion in other sectors (Miller et al., 2021). Moreover, ensembled mean projections, average per-country losses reaching 10.3% of agricultural output per year by 2091–2100 without considering mitigation strategies, and 4.5% with adaptation (Miller et al., 2021).

Breeding aims to become the main player in mitigating the effects of global warming. It was employed during the green revolution as a tool to boost yields by crossing smaller, hardier versions of common crops. Farmers used these alongside improved irrigation methods, strong pesticides and efficient fertilizers (Rehm, 2018). The cooperation of modelers, systems biologists, breeders, and farmers to accommodate environmental changes and improve sustainability, reflects the philosophy of the holistic approach needed to overcome the challenge involved in global warming.

Despite the continuous advances in plant science and understanding of the biophysical and molecular responses to local warming and temperature increase, little has been achieved to maintain crop yield and growth under temperature increases and to react to the consequent socio-economic challenges. It has been estimated that a breeding program takes about 30 years “from lab to fork” and although omics approaches have helped to reduce this time-scale, the interval between a discovery and its application is still too long (Varshney et al., 2014). Moreover, genetic breeding (molecular or not, engineered or not) mostly addresses individual traits, like resistance to a specific pathogen or pest, but is still poor in dealing with complex traits like tolerance to temperature increase (Comastri et al., 2018; Janni et al., 2020).

Thus, to address the global climate challenge a multifaceted and holistic approach in which crop production is seen only as one aspect of agroecosystem stress resilience is needed. To consider together the agroecosystem, the plant, and the novel technologies now available the shaping of more adaptable crops is mandatory.

The entire food chain, from the discovery of new varieties to their introduction in the market, requires suitable regulatory processes and distribution systems, which call for advanced management and marketing capacities. The entire chain that affects future developments has been termed the BDA process (Breeding, Delivery and Adoption) (Challinor et al., 2016). The means of adapting to global warming and temperature stress are certainly context-dependent, but they also show some common features. Knowledge-based strategies are needed to deal with food security both in developed and developing countries. In this field, the recent success of many African countries - the “African Green Revolution” - risks to being nullified by lack of strategies to help farmers overcome the problems posed by global warming.

Combination of “Omic” technologies are vital for the identification of key genes and metabolic pathways and can support marker-assisted breeding to cope with climate change (Zenda et al., 2021).The dissection of the genetic basis of important agronomic traits, as grain yield, grain size, flowering time, fiber quality and disease resistance paves the way for the application of new breeding techniques (NBTs) in breeding programs (Bohra et al., 2022) or in the exploitation of existing genetic resources through NGS (next-generation sequencing) (Mahmood et al., 2022). Moreover, plant phenotyping bridges two approaches essential for a sustainable production of food security: breeding and precision farming, both under controlled conditions (Janni and Pieruschka, 2022).

Campbell et al. (2016) (Campbell et al., 2016) pinpointed four challenges when counteracting the threats posed to food security by climate change: 1) changing the culture of research; 2) creating economical options for farmers, communities, and countries; 3) ensuring options that are relevant to the situations more affected by climate change; and 4) combining strategies such as adaptation and mitigation. Solutions like climate-change smart communities, and farming systems practicing Conservative Agriculture (Davies and Ribaut, 2017) are viewed with interest in developed countries too as permitting resilient agriculture and greater sustainability, and are well suited to the vision of a circular economy.

Climate change is in the process of imposing a highly selective extinction of animals and plants. Natural biodiversity alone does not suffice to preserve habitats and agroecosystems. It is obvious that human efforts will need to be directed to protect the low number of cultivated species essential for food security, by also exploring the existing biodiversity to discover novel alleles for climate adaptation (Danzi et al., 2021; Snowdon et al., 2021) and old species that may return useful. To address this emergency, more studies are explicitly considering complex and multifactorial stress combination (Dey et al., 2016; Lovell et al., 2016; Rivero et al., 2022; Zandalinas and Mittler, 2022). Thanks to these studies several evidence on the importance of higher level of complexity was found. While each of the different stresses (salt, high light, herbicides, heat, drought) applied individually, had a negligible effect on plant growth and survival, the accumulated impact of multifactorial stress combination on plants was detrimental. Unique and on that specific pathways and processes are triggered when combination of stresses was applied (Zandalinas and Mittler, 2022).

To exploit the molecular basis and processes associated with plant responses to HS, and the mechanisms of tolerance, more genome sequence information were essential including the pan-genomes of cultivated and wild species and precise identification of key alleles and genes. Precise identification and characterization of specific haplotypes will lay the foundation for genomic-assisted breeding strategies, including genome editing, for improved resilience, coupled with higher economic yields and higher sustainability.

To tackle the upcoming HS scenarios, a new breeding paradigm is required to focus not on single stress effectors but to move in the direction of higher complexity. The adoption of a holistic approach for climate-resilient breeding should be the next revolution to enable the sustainability of crop production.

Sustainability goes beyond three precise steps within the food supply chain: i) development of food systems; ii) reduction of food loss and waste (FLW); and iii) global dietary change toward plant-based diets (Garcia-Oliveira et al., 2022).

The holistic approach starts from considering the trade-off between food security and nutrition; livelihoods; environmental sustainability, novel technology. The proposed approach meets the targets of the Sustainable Development Goals (SDGs) - in particular SDG 2, which aims to create a world free of hunger by 2030. Again, the integration of socioeconomic developments and climatic crisis within the context of global change and worst the need to prompt policymakers and stakeholders to consider these insights to inform future assessments and policymaking efforts.

Adaptation to climate change of agroecosystems requires holistic actions and the shift from punctual responses to an integrated approach but on the same scale. Some proposals in this direction are related to technical interventions, for example, from genomic and phenotypic characterization to obtain seed varieties that were more resistant to drought and high temperatures, varieties with adapted growth cycles, modifications on the use of agricultural amendments, and optimization of precision irrigation methods (Mirón et al., 2023).

In view of a holistic approach resource savings technologies should be considered as mitigating technology toward the achievement of increased sustainability (Ermakova et al., 2021).

Precision agriculture technologies have the potential to play a key role in the implementation of Climate Smart Agriculture by aiding farmers to tailor farm inputs and management conditions (Toriyama, 2020). Several key technologies are already in use in agriculture to improve sustainability and resource use efficiency as for example variable rate application that allowed for a strong reduction in N_2_O usage up to 34% (Mamo et al., 2003; Kanter et al., 2019).

Irrigation, as the use of special multilayer soil structures (fertile layer/hydro accumulating layer/sand), secondary water for irrigation, and desalination of salt water, using reverse osmosis or evaporation, embracing the concept of circular economy as part of the global solution (Myrzabaeva et al., 2017; Martinez-Alvarez et al., 2020; Gao et al., 2022). But how to mitigate climate change from a circularity perspective has become a trending topic (Romero-Perdomo et al., 2022) more than a search for pragmatic solutions.

In this frame, novel technologies based sensors as remote, proximal and *in vivo* sensors and sensor’s platforms can significantly enhance irrigation efficiency and produce water savings (Janni et al., 2019; Segarra et al., 2020; Tavan et al., 2021; Kim and Lee, 2022) becoming more familiar in everyday farm management.

Finally, and ironically, the omics approach has generated data which emphasizes epigenetics, the broad term used to describe all causes of variation which cannot be explained with classical genetics. Transposons, non-coding RNAs, chromatin regulation and chemical modification are among these. One point of considerable interest is the role of non-coding RNAs such as microRNA(miRNA) in modulating plant response to several abiotic stresses including HS (Pagano et al., 2021), and the fact that these miRNAs are part of the innate reaction to this stress, the “plant immune system”.

This work is aimed at opening new perspectives for dissemination and to give novel thoughts in the light to mitigate the dramatic effects of climate change. Overall, the holistic approach targets several areas of interest to public research institutions, policy makers, food producers and farmers, brad public, and consumers. Omics in this vision, represents a first and road in the sustainability in agriculture (Braun et al., 2023; Gil, 2023). This work considers all aspects of food production, highlighting the strength and weak of the current approaches.

## Author contributions

MJ: Conceptualization, Writing – original draft. EM: Supervision, Writing – review & editing. MG: Supervision, Writing – review & editing. MM: Supervision, Writing – review & editing. NM: Funding acquisition, Supervision, Writing – original draft.

## References

[B1] Abdel-Aziz H. M. M. Soliman M. I. Abo Al-Saoud A. M. El-Sherbeny G. A. (2021). Waste-derived NPK nanofertilizer enhances growth and productivity of capsicum annuum L. Plants 10, 1144. doi: 10.3390/plants10061144 34199718 PMC8227464

[B2] Ali A. S. Elozeiri A. A. Ali A. S. Elozeiri A. A. (2017). “Metabolic processes during seed germination,” in Advances in seed biology (IntechOpen). doi: 10.5772/intechopen.70653

[B3] Allan C. Nguyen T. P. L. Seddaiu G. Wilson B. Roggero P. P. (2013). Integrating local knowledge with experimental research: case studies on managing cropping systems in Italy and Australia. Ital. J. Agron. 8, e15–e15. doi: 10.4081/ija.2013.e15

[B4] Alom M. Z. Taha T. M. Yakopcic C. Westberg S. Sidike P. Nasrin M. S.. (2019). A state-of-the-art survey on deep learning theory and architectures. Electronics 8, 292. doi: 10.3390/electronics8030292

[B5] Araus J. L. Kefauver S. C. (2018). Breeding to adapt agriculture to climate change: affordable phenotyping solutions. Curr. Opin. Plant Biol 13, 1–11. doi: 10.1016/j.pbi.2018.05.003 29853283

[B6] Araus J. L. Kefauver S. C. Zaman-Allah M. Olsen M. S. Cairns J. E. (2018). Translating high-throughput phenotyping into genetic gain. Trends Plant Sci. 23, 451–466. doi: 10.1016/j.tplants.2018.02.001 29555431 PMC5931794

[B7] Backer R. Rokem J. S. Ilangumaran G. Lamont J. Praslickova D. Ricci E.. (2018). Plant growth-promoting rhizobacteria: context, mechanisms of action, and roadmap to commercialization of biostimulants for sustainable agriculture. Front. Plant Sci. 9. doi: 10.3389/fpls.2018.01473 PMC6206271 30405652

[B8] Barghi S. S. Mostafaii H. Peighami F. Zakaria R. A. (2012). PATH ANALYSIS OF YIELD AND ITS COMPONENTS IN LENTIL UNDER END SEASON HEAT CONDITION.

[B9] Beesley L. Moreno-Jiménez E. Gomez-Eyles J. L. (2010). Effects of biochar and greenwaste compost amendments on mobility, bioavailability and toxicity of inorganic and organic contaminants in a multi-element polluted soil. Environ. pollut. 158, 2282–2287. doi: 10.1016/j.envpol.2010.02.003 20219274

[B10] Bhardwaj D. Ansari M. W. Sahoo R. K. Tuteja N. (2014). Biofertilizers function as key player in sustainable agriculture by improving soil fertility, plant tolerance and crop productivity. Microb. Cell Factories 13, 66. doi: 10.1186/1475-2859-13-66 PMC4022417 24885352

[B11] Bhattacharya A. (2019). Effect of high temperature on crop productivity and metabolism of macro molecules (Academic Press).

[B12] Bhupenchandra I. Chongtham S. K. Devi E. L. R. R. Choudhary A. K. Salam M. D.. (2022). Role of biostimulants in mitigating the effects of climate change on crop performance. Front. Plant Sci. 13. doi: 10.3389/fpls.2022.967665 PMC9634556 36340395

[B13] Bohra A. Kilian B. Sivasankar S. Caccamo M. Mba C. McCouch S. R.. (2022). Reap the crop wild relatives for breeding future crops. Trends Biotechnol. 40, 412–431. doi: 10.1016/j.tibtech.2021.08.009 34629170

[B14] Brás T. A. Seixas J. Carvalhais N. Jägermeyr J. (2021). Severity of drought and heatwave crop losses tripled over the last five decades in Europe. Environ. Res. Lett 16, 065012. doi: 10.1088/1748-9326/abf004

[B15] Braun D. M. Washburn J. D. Wood J. D. (2023). Enhancing the resilience of plant systems to climate change. J. Exp. Bot. 74, 2787–2789. doi: 10.1093/jxb/erad090 37103001

[B16] Broecker W. S. (1975). Climatic change: are we on the brink of a pronounced global warming? Science 189, 460–463. doi: 10.1126/science.189.4201.460 17781884

[B17] Brower-Toland B. Shyu C. Vega-Sanchez M. E. Slewinski T. L. (2023). Pedigree or identity? How genome editing can fundamentally change the path for crop development. J. Exp. Bot. 74, 2794–2798. doi: 10.1093/jxb/erad033 36738269 PMC10134896

[B18] Buffagni V. Vurro F. Janni M. Gullì M. Keller A. A. Marmiroli N. (2020). Shaping durum wheat for the future: gene expression analyses and metabolites profiling support the contribution of BCAT genes to drought stress response. Front. Plant Sci. 11. doi: 10.3389/fpls.2020.00891 PMC7350509 32719694

[B19] Cabell J. Oelofse M. (2012). An indicator framework for assessing agroecosystem resilience. Ecol. Soc 17 (1), 18. doi: 10.5751/ES-04666-170118

[B20] Campbell B. M. Vermeulen S. J. Aggarwal P. K. Corner-Dolloff C. Girvetz E. Loboguerrero A. M.. (2016). Reducing risks to food security from climate change. Glob. Food Secur. 11, 34–43. doi: 10.1016/j.gfs.2016.06.002

[B21] Carpenter S. Walker B. Anderies J. M. Abel N. (2001). From metaphor to measurement: resilience of what to what? Ecosystems 4, 765–781. doi: 10.1007/s10021-001-0045-9

[B22] Chakraborty S. Newton A. C. (2011). Climate change, plant diseases and food security: an overview. Plant Pathol. 60, 2–14. doi: 10.1111/j.1365-3059.2010.02411.x

[B23] Challinor A. J. Koehler A.-K. Ramirez-Villegas J. Whitfield S. Das B. (2016). Current warming will reduce yields unless maize breeding and seed systems adapt immediately. Nat. Clim. Change 6, 954–958. doi: 10.1038/nclimate3061

[B24] Challinor A. J. Watson J. Lobell D. B. Howden S. M. Smith D. R. Chhetri N. (2014). A meta-analysis of crop yield under climate change and adaptation. Nat. Clim. Change 4, 287–291. doi: 10.1038/nclimate2153

[B25] Chaturvedi P. Wiese A. J. Ghatak A. Záveská Drábková L. Weckwerth W. Honys D. (2021). Heat stress response mechanisms in pollen development. New Phytol. 231, 571–585. doi: 10.1111/nph.17380 33818773 PMC9292940

[B26] Comastri A. Janni M. Simmonds J. Uauy C. Pignone D. Nguyen H. T.. (2018). Heat in wheat: exploit reverse genetic techniques to discover new alleles within the Triticum durum sHsp26 family. Front. Plant Sci. 9. doi: 10.3389/fpls.2018.01337 PMC6156267 30283469

[B27] Coppedè N. Janni M. Bettelli M. Maida C. L. Gentile F. Villani M.. (2017). An in *vivo* biosensing, biomimetic electrochemical transistor with applications in plant science and precision farming. Sci. Rep. 7, 16195. doi: 10.1038/s41598-017-16217-4 29170393 PMC5700984

[B28] Danzi D. Briglia N. Petrozza A. Summerer S. Povero G. Stivaletta A.. (2019). Can high throughput phenotyping help food security in the mediterranean area? Front. Plant Sci. 10. doi: 10.3389/fpls.2019.00015 PMC6355677 30740116

[B29] Danzi D. Marino I. De Bari I. Mastrolitti S. Petretto G. L. Pignone D.. (2021). Assessment of durum wheat (Triticum durum desf.) genotypes diversity for the integrated production of bioethanol and grains. Energies 14, 7735. doi: 10.3390/en14227735

[B30] David E. Madec S. Sadeghi-Tehran P. Aasen H. Zheng B. Liu S.. (2020). Global wheat head detection (GWHD) dataset: A large and diverse dataset of high-resolution RGB-labelled images to develop and benchmark wheat head detection methods. Plant Phenomics 2020, 3521852. doi: 10.34133/2020/3521852 33313551 PMC7706323

[B31] Davies W. J. Ribaut J.-M. (2017). Stress resilience in crop plants: strategic thinking to address local food production problems. Food Energy Secur. 6, 12–18. doi: 10.1002/fes3.105

[B32] Degener J. F. (2015). Atmospheric CO2 fertilization effects on biomass yields of 10 crops in northern Germany. Front. Environ. Sci. 3. doi: 10.3389/fenvs.2015.00048

[B33] de Lima C. Z. Buzan J. R. Moore F. C. Baldos U. L. C. Huber M. Hertel T. W. (2021). Heat stress on agricultural workers exacerbates crop impacts of climate change. Environ. Res. Lett. 16, 044020. doi: 10.1088/1748-9326/abeb9f

[B34] Deutsch C. A. Tewksbury J. J. Tigchelaar M. Battisti D. S. Merrill S. C. Huey R. B.. (2018). Increase in crop losses to insect pests in a warming climate. Science 361, 916–919. doi: 10.1126/science.aat3466 30166490

[B35] Dey S. Proulx S. R. Teotónio H. (2016). Adaptation to temporally fluctuating environments by the evolution of maternal effects. PloS Biol. 14, e1002388. doi: 10.1371/journal.pbio.1002388 26910440 PMC4766184

[B36] Di Falco S. Chavas J.-P. (2008). Rainfall shocks, resilience, and the effects of crop biodiversity on agroecosystem productivity. Land Econ. 84, 83–96. doi: 10.3368/le.84.1.83

[B37] Driedonks N. Rieu I. Vriezen W. H. (2016). Breeding for plant heat tolerance at vegetative and reproductive stages. Plant Reprod. 29, 67–79. doi: 10.1007/s00497-016-0275-9 26874710 PMC4909801

[B38] Ermakova A. M. Demina K. A. Nurullina T. S. (2021). “Resource-saving technologies - the basis of effective enterprise activity,” in IOP Conf. Ser. Earth Environ. Sci, Vol. 723. 042027. doi: 10.1088/1755-1315/723/4/042027

[B39] Fahad S. Bajwa A. A. Nazir U. Anjum S. A. Farooq A. Zohaib A.. (2017). Crop production under drought and heat stress: plant responses and management options. Front. Plant Sci. 8. doi: 10.3389/fpls.2017.01147 PMC5489704 28706531

[B40] FAO (2020). The state of food and agriculture 2020 (FAO). doi: 10.4060/cb1447en

[B41] Fiorani F. Schurr U. (2013). Future scenarios for plant phenotyping. Annu. Rev. Plant Biol. 64, 267–291. doi: 10.1146/annurev-arplant-050312-120137 23451789

[B42] Foley J. A. Ramankutty N. Brauman K. A. Cassidy E. S. Gerber J. S. Johnston M.. (2011). Solutions for a cultivated planet. Nature 478, 337–342. doi: 10.1038/nature10452 21993620

[B43] Foyer C. H. Lam H.-M. Nguyen H. T. Siddique K. H. M. Varshney R. K. Colmer T. D.. (2016). Neglecting legumes has compromised human health and sustainable food production. Nat. Plants 2, 16112. doi: 10.1038/nplants.2016.112 28221372

[B44] Gao H. Guo R. Shi K. Yue H. Zu S. Li Z.. (2022). Effect of different water treatments in soil-plant-atmosphere continuum based on intelligent weighing systems. Water 14, 673. doi: 10.3390/w14040673

[B45] Gao G. Tester M. A. Julkowska M. M. (2020). The use of high-throughput phenotyping for assessment of heat stress-induced changes in arabidopsis. Plant Phenomics 14 (4). doi: 10.34133/2020/3723916 PMC7706305 33313552

[B46] Garcia-Oliveira P. Fraga-Corral M. Carpena M. Prieto M. A. Simal-Gandara J. (2022). “Chapter 2 - Approaches for sustainable food production and consumption systems,” in Future foods. Ed. Bhat R. (Academic Press), 23–38. doi: 10.1016/B978-0-323-91001-9.00006-2

[B47] Gil J. (2023). Forgotten crops confer resilience under climate change. Nat. Food 4, 275–275. doi: 10.1038/s43016-023-00754-5

[B48] Graziano S. Caldara M. Gullì M. Bevivino A. Maestri E. Marmiroli N. (2022). A Metagenomic and Gene Expression Analysis in Wheat (T. durum) and Maize (Z. mays) Biofertilized with PGPM and Biochar. Int. J. Mol. Sci. 23, 10376. doi: 10.3390/ijms231810376 36142289 PMC9499264

[B49] Han S. Jiang S. Xiong R. Shafique K. Zahid K. R. Wang Y. (2022). Response and tolerance mechanism of food crops under high temperature stress: a review. Braz. J. Biol. 82, e253898. doi: 10.1590/1519-6984.253898 35107484

[B50] Holling C. S. (1978). “Myths of ecological stability: Resilience and the problem of failure,” in Studies in crisis management.

[B51] Holling C. S. (2001). Understanding the complexity of economic, ecological, and social systems. Ecosystems 4, 390–405. doi: 10.1007/s10021-001-0101-5

[B52] Hoy C. (2015). Agroecosystem health, agroecosystem resilience, and food security. J. Environ. Stud. Sci. 5, 623–635. doi: 10.1007/s13412-015-0322-0

[B53] Huang X.-Y. Salt D. E. (2016). Plant ionomics: from elemental profiling to environmental adaptation. Mol. Plant 9, 787–797. doi: 10.1016/j.molp.2016.05.003 27212388

[B54] Hurkman W. J. McCue K. F. Altenbach S. B. Korn A. Tanaka C. K. Kothari K. M.. (2003). Effect of temperature on expression of genes encoding enzymes for starch biosynthesis in developing wheat endosperm. Plant Sci. 164, 873–881. doi: 10.1016/S0168-9452(03)00076-1

[B55] Hurkman W. J. Tanaka C. K. Vensel W. H. Thilmony R. Altenbach S. B. (2013). Comparative proteomic analysis of the effect of temperature and fertilizer on gliadin and glutenin accumulation in the developing endosperm and flour from Triticum aestivum L. cv. Butte 86. Proteome Sci. 11, 8. doi: 10.1186/1477-5956-11-8 23432757 PMC3599944

[B56] IPCC (2019). Climate change and land.

[B57] Janni M. Coppede N. Bettelli M. Briglia N. Petrozza A. Summerer S.. (2019). *In vivo* phenotyping for the early detection of drought stress in tomato. Plant Phenomics 2019, 1–10. doi: 10.34133/2019/6168209 PMC7706337 33313533

[B58] Janni M. Gullì M. Maestri E. Marmiroli M. Valliyodan B. Nguyen H. T.. (2020). Molecular and genetic bases of heat stress responses in crop plants and breeding for increased resilience and productivity. J. Exp. Bot. 71, 3780–3802. doi: 10.1093/jxb/eraa034 31970395 PMC7316970

[B59] Janni M. Pieruschka R. (2022). Plant phenotyping for a sustainable future. J. Exp. Bot. 73, 5085–5088. doi: 10.1093/jxb/erac286 36056763

[B60] Juliana P. Montesinos-López O. A. Crossa J. Mondal S. González Pérez L. Poland J.. (2019). Integrating genomic-enabled prediction and high-throughput phenotyping in breeding for climate-resilient bread wheat. Theor. Appl. Genet. 132, 177–194. doi: 10.1007/s00122-018-3206-3 30341493 PMC6320358

[B61] Kah M. Kookana R. S. Gogos A. Bucheli T. D. (2018). A critical evaluation of nanopesticides and nanofertilizers against their conventional analogues. Nat. Nanotechnol. 13, 677–684. doi: 10.1038/s41565-018-0131-1 29736032

[B62] Kah M. Tufenkji N. White J. C. (2019). Nano-enabled strategies to enhance crop nutrition and protection. Nat. Nanotechnol. 14, 532–540. doi: 10.1038/s41565-019-0439-5 31168071

[B63] Kalwani M. Chakdar H. Srivastava A. Pabbi S. Shukla P. (2022). Effects of nanofertilizers on soil and plant-associated microbial communities: Emerging trends and perspectives. Chemosphere 287, 132107. doi: 10.1016/j.chemosphere.2021.132107 34492409

[B64] Kang Y. Lee K. Hoshikawa K. Kang M. Jang S. (2022). Molecular bases of heat stress responses in vegetable crops with focusing on heat shock factors and heat shock proteins. Front. Plant Sci. 13. doi: 10.3389/fpls.2022.837152 PMC9036485 35481144

[B65] Kanter D. R. Bell A. R. McDermid S. S. (2019). Precision agriculture for smallholder nitrogen management. One Earth 1, 281–284. doi: 10.1016/j.oneear.2019.10.015

[B66] Karwa S. Bahuguna R. N. Chaturvedi A. K. Maurya S. Arya S. S. Chinnusamy V.. (2020). Phenotyping and characterization of heat stress tolerance at reproductive stage in rice (Oryza sativa L.). Acta Physiol. Plant 42, 29. doi: 10.1007/s11738-020-3016-5

[B67] Katam R. Shokri S. Murthy N. Singh S. K. Suravajhala P. Khan M. N.. (2020). Proteomics, physiological, and biochemical analysis of cross tolerance mechanisms in response to heat and water stresses in soybean. PloS One 15 (6), e0233905. doi: 10.1371/journal.pone.0233905 32502194 PMC7274410

[B68] Kaushal N. Bhandari K. Siddique K. H. M. Nayyar H. (2016). Food crops face rising temperatures: An overview of responses, adaptive mechanisms, and approaches to improve heat tolerance. Cogent Food Agric. 2, 1134380. doi: 10.1080/23311932.2015.1134380

[B69] Kemp L. Xu C. Depledge J. Ebi K. L. Gibbins G. Kohler T. A.. (2022). Climate Endgame: Exploring catastrophic climate change scenarios. Proc. Natl. Acad. Sci. 119, e2108146119. doi: 10.1073/pnas.2108146119 35914185 PMC9407216

[B70] Khoury C. K. Bjorkman A. D. Dempewolf H. Ramirez-Villegas J. Guarino L. Jarvis A.. (2014). Increasing homogeneity in global food supplies and the implications for food security. Proc. Natl. Acad. Sci. 111, 4001–4006. doi: 10.1073/pnas.1313490111 24591623 PMC3964121

[B71] Kim M.-Y. Lee K. H. (2022). Electrochemical sensors for sustainable precision agriculture—A review. Front. Chem. 10. doi: 10.3389/fchem.2022.848320 PMC9124781 35615311

[B72] Konduri V. S. Vandal T. J. Ganguly S. Ganguly A. R. (2020). Data science for weather impacts on crop yield. Front. Sustain. Food Syst. 4. doi: 10.3389/fsufs.2020.00052

[B73] Kumar L. Chhogyel N. Gopalakrishnan T. Hasan M. K. Jayasinghe S. L. Kariyawasam C. S.. (2022). “Chapter 4 - Climate change and future of agri-food production,” in Future foods. Ed. Bhat R. (Academic Press), 49–79. doi: 10.1016/B978-0-323-91001-9.00009-8

[B74] Kumar R. R. Singh K. Ahuja S. Tasleem M. Singh I. Kumar S.. (2019). Quantitative proteomic analysis reveals novel stress-associated active proteins (SAAPs) and pathways involved in modulating tolerance of wheat under terminal heat. Funct. Integr. Genomics 19, 329–348. doi: 10.1007/s10142-018-0648-2 30465139

[B75] Law B. E. Hudiburg T. W. Berner L. T. Kent J. J. Buotte P. C. Harmon M. E. (2018). Land use strategies to mitigate climate change in carbon dense temperate forests. Proc. Natl. Acad. Sci. 115, 3663–3668. doi: 10.1073/pnas.1720064115 29555758 PMC5889652

[B76] Li S. Chan C. Y. (2022). Will biochar suppress or stimulate greenhouse gas emissions in agricultural fields? Unveiling the dice game through data syntheses. Soil Syst. 6, 73. doi: 10.3390/soilsystems6040073

[B77] Li Y. Yu Z. Jin J. Zhang Q. Wang G. Liu C.. (2018). Impact of elevated CO2 on seed quality of soybean at the fresh edible and mature stages. Front. Plant Sci. 9. doi: 10.3389/fpls.2018.01413 PMC6199416 30386351

[B78] Li L. Zhang Q. Huang D. (2014). A review of imaging techniques for plant phenotyping. Sensors 14, 20078–20111. doi: 10.3390/s141120078 25347588 PMC4279472

[B79] Liu T. Zhang X. Li K. Yao Q. Zhong D. Deng Q.. (2023). Large-scale genome editing in plants: approaches, applications, and future perspectives. Curr. Opin. Biotechnol. 79, 102875. doi: 10.1016/j.copbio.2022.102875 36610369

[B80] Lohani N. Singh M. B. Bhalla P. L. (2020). High temperature susceptibility of sexual reproduction in crop plants. J. Exp. Bot. 71, 555–568. doi: 10.1093/jxb/erz426 31560053

[B81] Long S. P. Ainsworth E. A. Leakey A. D. B. Nösberger J. Ort D. R. (2006). Food for thought: lower-than-expected crop yield stimulation with rising CO2 concentrations. Science 312, 1918–1921. doi: 10.1126/science.1114722 16809532

[B82] Lopes M. J. dos S. Dias-Filho M. B. Gurgel E. S. C. (2021). Successful plant growth-promoting microbes: inoculation methods and abiotic factors. Front. Sustain. Food Syst. 5. doi: 10.3389/fsufs.2021.606454

[B83] Lovell J. T. Shakirov E. V. Schwartz S. Lowry D. B. Aspinwall M. J. Taylor S. H.. (2016). Promises and challenges of eco-physiological genomics in the field: tests of drought responses in switchgrass1[OPEN]. Plant Physiol. 172, 734–748. doi: 10.1104/pp.16.00545 27246097 PMC5047078

[B84] Luan X. Vico G. (2021). Canopy temperature and heat stress are increased by compound high air temperature and water stress and reduced by irrigation – a modeling analysis. Hydrol. Earth Syst. Sci. 25, 1411–1423. doi: 10.5194/hess-25-1411-2021

[B85] Maestri E. Klueva N. Perrotta C. Gulli M. Nguyen H. T. Marmiroli N. (2002). Molecular genetics of heat tolerance and heat shock proteins in cereals. Plant Mol. Biol. 48, 667–681. doi: 10.1023/A:1014826730024 11999842

[B86] Mahmood U. Li X. Fan Y. Chang W. Niu Y. Li J.. (2022). Multi-omics revolution to promote plant breeding efficiency. Front. Plant Sci. 13. doi: 10.3389/fpls.2022.1062952 PMC9773847 36570904

[B87] Malhi G. S. Kaur M. Kaushik P. (2021). Impact of climate change on agriculture and its mitigation strategies: A review. Sustainability 13, 1318. doi: 10.3390/su13031318

[B88] Mamo M. Malzer G. L. Mulla D. J. Huggins D. R. Strock J. (2003). Spatial and temporal variation in economically optimum nitrogen rate for corn. Agron. J. 95, 958–964. doi: 10.2134/agronj2003.9580

[B89] Mangelsen E. Kilian J. Harter K. Jansson C. Wanke D. Sundberg E. (2011). Transcriptome analysis of high-temperature stress in developing barley caryopses: early stress responses and effects on storage compound biosynthesis. Mol. Plant 4, 97–115. doi: 10.1093/mp/ssq058 20924027

[B90] Marko D. El-shershaby A. Carriero F. Summerer S. Petrozza A. Iannacone R.. (2019). Identification and characterization of a thermotolerant TILLING allele of heat shock binding protein 1 in tomato. Genes 10, 516. doi: 10.3390/genes10070516 31284688 PMC6678839

[B91] Marmiroli M. Caldara M. Pantalone S. Malcevschi A. Maestri E. Keller A. A.. (2022). Building a risk matrix for the safety assessment of wood derived biochars. Sci. Total Environ. 839, 156265. doi: 10.1016/j.scitotenv.2022.156265 35643132

[B92] Marmiroli M. Pagano L. Rossi R. de la Torre-Roche R. Lepore G. O. Ruotolo R.. (2021). Copper oxide nanomaterial fate in plant tissue: nanoscale impacts on reproductive tissues. Environ. Sci. Technol. 55, 10769–10783. doi: 10.1021/acs.est.1c01123 34308629

[B93] Martinez-Alvarez V. Bar-Tal A. Diaz Peña F. J. Maestre-Valero J. F. (2020). Desalination of seawater for agricultural irrigation. Water 12, 1712. doi: 10.3390/w12061712

[B94] Masouleh S. S. S. Sassine Y. N. (2020). Molecular and biochemical responses of horticultural plants and crops to heat stress. Ornam. Hortic. 26, 148–158. doi: 10.1590/2447-536X.v26i2.2134

[B95] Miller S. Chua K. Coggins J. Mohtadi H. (2021). Heat waves, climate change, and economic output. J. Eur. Econ. Assoc. 19, 2658–2694. doi: 10.1093/jeea/jvab009

[B96] Mirón I. J. Linares C. Díaz J. (2023). The influence of climate change on food production and food safety. Environ. Res. 216, 114674. doi: 10.1016/j.envres.2022.114674 36341795

[B97] Mohamed M. F. Thalooth A. T. Elewa T. A. Ahmed A. G. (2019). Yield and nutrient status of wheat plants (Triticum aestivum) as affected by sludge, compost, and biofertilizers under newly reclaimed soil. Bull. Natl. Res. Cent. 43, 31. doi: 10.1186/s42269-019-0069-y

[B98] Mote G. Jadhav P. Magar S. Thakur P. Moharil M. Biradar R. (2022). “CRISPR/cas-based genome editing to enhance heat stress tolerance in crop plants,” in Thermotolerance in crop plants. Eds. Kumar R. R. Praveen S. Rai G. K. (Springer Nature), 281–297. doi: 10.1007/978-981-19-3800-9_13

[B99] Muthamilarasan M. Singh N. K. Prasad M. (2019). Multi-omics approaches for strategic improvement of stress tolerance in underutilized crop species: A climate change perspective. Adv. Genet. 103, 1–38. doi: 10.1016/bs.adgen.2019.01.001 30904092

[B100] Myrzabaeva M. Insepov Z. Boguspaev K. Faleev D. Nazhipkyzy M. Lesbayev B.. (2017). Investigation of nanohydrophobic sand as an insulating layer for cultivation of plants in soils contaminated with heavy metals. Eurasian Chem.-Technol. J. 19, 91. doi: 10.18321/ectj507

[B101] Narayanan S. Prasad P. V. V. Welti R. (2016). Wheat leaf lipids during heat stress: II. Lipids experiencing coordinated metabolism are detected by analysis of lipid co-occurrence. Plant Cell Environ. 39, 608–617. doi: 10.1111/pce.12648 26436445 PMC5141584

[B102] Narayanan S. Prasad P. V. V. Welti R. (2018). Alterations in wheat pollen lipidome during high day and night temperature stress: Heat induced alterations in wheat pollen lipidome. Plant Cell Environ. 41, 1749–1761. doi: 10.1111/pce.13156 29377219 PMC6713575

[B103] Pagano L. Rossi R. Paesano L. Marmiroli N. Marmiroli M. (2021). miRNA regulation and stress adaptation in plants. Environ. Exp. Bot. 184, 104369. doi: 10.1016/j.envexpbot.2020.104369

[B104] Pareek A. Dhankher O. P. Foyer C. H. (2020). Mitigating the impact of climate change on plant productivity and ecosystem sustainability. J. Exp. Bot. 71, 451–456. doi: 10.1093/jxb/erz518 31909813 PMC6945998

[B105] Parthasarathi T. Firdous S. David E. M. Lesharadevi K. Djanaguiraman M. Parthasarathi T.. (2022). Effects of high temperature on crops (IntechOpen). doi: 10.5772/intechopen.105945

[B106] Paupière M. J. van Haperen P. Rieu I. Visser R. G. F. Tikunov Y. M. Bovy A. G. (2017). Screening for pollen tolerance to high temperatures in tomato. Euphytica 213, 130. doi: 10.1007/s10681-017-1927-z

[B107] Pavlicevic M. Abdelraheem W. Zuverza-Mena N. O’Keefe T. Mukhtar S. Ridge G.. (2022). Engineered nanoparticles, natural nanoclay and biochar, as carriers of plant-growth promoting bacteria. Nanomaterials 12, 4474. doi: 10.3390/nano12244474 36558327 PMC9783841

[B108] Perez-Sanz F. Navarro P. J. Egea-Cortines M. (2017). Plant phenomics: an overview of image acquisition technologies and image data analysis algorithms. GigaScience 6, 1–18. doi: 10.1093/gigascience/gix092 PMC5737281 29048559

[B109] Petrozza A. Santaniello A. Summerer S. Di Tommaso G. Di Tommaso D. Paparelli E.. (2014). Physiological responses to Megafol^®^ treatments in tomato plants under drought stress: A phenomic and molecular approach. Sci. Hortic. 174, 185–192. doi: 10.1016/j.scienta.2014.05.023

[B110] Pettenuzzo S. Cappellin L. Grando M. S. Costantini L. (2022). Phenotyping methods to assess heat stress resilience in grapevine. J. Exp. Bot. 73, 5128–5148. doi: 10.1093/jxb/erac058 35532318

[B111] Pita-Barbosa A. Ricachenevsky F. K. Flis P. M. (2019). One “OMICS” to integrate them all: ionomics as a result of plant genetics, physiology and evolution. Theor. Exp. Plant Physiol. 31, 71–89. doi: 10.1007/s40626-019-00144-y

[B112] Prasad P. V. V. Djanaguiraman M. (2014). Response of floret fertility and individual grain weight of wheat to high temperature stress: sensitive stages and thresholds for temperature and duration. Funct. Plant Biol. 41, 1261–1269. doi: 10.1071/FP14061 32481075

[B113] Priya M. Dhanker O. P. Siddique K. H. M. HanumanthaRao B. Nair R. M. Pandey S.. (2019). Drought and heat stress-related proteins: an update about their functional relevance in imparting stress tolerance in agricultural crops. Theor. Appl. Genet. 132, 1607–1638. doi: 10.1007/s00122-019-03331-2 30941464

[B114] Qu M. Chen G. Bunce J. A. Zhu X. Sicher R. C. (2018). Systematic biology analysis on photosynthetic carbon metabolism of maize leaf following sudden heat shock under elevated CO2. Sci. Rep. 8, 7849. doi: 10.1038/s41598-018-26283-x 29777170 PMC5959914

[B115] Ramakrishnan B. Maddela N. R. Venkateswarlu K. Megharaj M. (2021). Organic farming: Does it contribute to contaminant-free produce and ensure food safety? Sci. Total Environ. 769, 145079. doi: 10.1016/j.scitotenv.2021.145079 33482543

[B116] Raza A. Razzaq A. Mehmood S. S. Hussain M. A. Wei S. He H.. (2021a). Omics: The way forward to enhance abiotic stress tolerance in Brassica napus L. GM Crops Food 12, 251–281. doi: 10.1080/21645698.2020.1859898 33464960 PMC7833762

[B117] Raza A. Tabassum J. Kudapa H. Varshney R. K. (2021b). Can omics deliver temperature resilient ready-to-grow crops? Crit. Rev. Biotechnol. 41 (8), 1209–1232. doi: 10.1080/07388551.2021.1898332 33827346

[B118] Rehm J. (2018). Green revolution’ crops bred to slash fertilizer use. Nature. doi: 10.1038/d41586-018-05980-7

[B119] Ribeiro A. F. S. Russo A. Gouveia C. M. Páscoa P. Zscheischler J. (2020). Risk of crop failure due to compound dry and hot extremes estimated with nested copulas. Biogeophysics: Ecohydrology 17, 4815–4830. doi: 10.5194/bg-2020-116

[B120] Rivero R. M. Mittler R. Blumwald E. Zandalinas S. I. (2022). Developing climate-resilient crops: improving plant tolerance to stress combination. Plant J. 109, 373–389. doi: 10.1111/tpj.15483 34482588

[B121] Rombel A. Krasucka P. Oleszczuk P. (2022). Sustainable biochar-based soil fertilizers and amendments as a new trend in biochar research. Sci. Total Environ. 816, 151588. doi: 10.1016/j.scitotenv.2021.151588 34774939

[B122] Romero-Perdomo F. Carvajalino-Umaña J. D. Moreno-Gallego J. L. Ardila N. González-Curbelo M.Á. (2022). Research trends on climate change and circular economy from a knowledge mapping perspective. Sustainability 14, 521. doi: 10.3390/su14010521

[B123] Rouphael Y. Colla G. (2020). Editorial: biostimulants in agriculture. Front. Plant Sci. 11. doi: 10.3389/fpls.2020.00040 PMC7010726 32117379

[B124] Sadok W. Jagadish S. V. K. (2020). The hidden costs of nighttime warming on yields. Trends Plant Sci. 25, 644–651. doi: 10.1016/j.tplants.2020.02.003 32526169

[B125] Saeed F. Chaudhry U. K. Raza A. Charagh S. Bakhsh A. Bohra A.. (2023). Developing future heat-resilient vegetable crops. Funct. Integr. Genomics 23, 47. doi: 10.1007/s10142-023-00967-8 36692535 PMC9873721

[B126] Sangiorgio D. Cellini A. Donati I. Pastore C. Onofrietti C. Spinelli F. (2020). Facing climate change: application of microbial biostimulants to mitigate stress in horticultural crops. Agronomy 10, 794. doi: 10.3390/agronomy10060794

[B127] Scharf K.-D. Berberich T. Ebersberger I. Nover L. (2012). The plant heat stress transcription factor (Hsf) family: Structure, function and evolution. Biochim. Biophys. Acta BBA - Gene Regul. Mech. 1819, 104–119. doi: 10.1016/j.bbagrm.2011.10.002 22033015

[B128] Schauberger G. Schönhart M. Zollitsch W. Hörtenhuber S. J. Kirner L. Mikovits C.. (2021). Economic risk assessment by weather-related heat stress indices for confined livestock buildings: A case study for fattening pigs in central europe. Agriculture 11, 122. doi: 10.3390/agriculture11020122

[B129] Schwachtje J. Whitcomb S. J. Firmino A. A. P. Zuther E. Hincha D. K. Kopka J. (2019). Induced, imprinted, and primed responses to changing environments: does metabolism store and process information? Front. Plant Sci. 10. doi: 10.3389/fpls.2019.00106 PMC6381073 30815006

[B130] Segarra J. Buchaillot M. L. Araus J. L. Kefauver S. C. (2020). Remote sensing for precision agriculture: sentinel-2 improved features and applications. Agronomy 10, 641. doi: 10.3390/agronomy10050641

[B131] Sehgal A. Sita K. Kumar J. Kumar S. Singh S. Siddique K. H. M.. (2017). Effects of drought, heat and their interaction on the growth, yield and photosynthetic function of lentil (Lens culinaris medikus) genotypes varying in heat and drought sensitivity. Front. Plant Sci. 8. doi: 10.3389/fpls.2017.01776 PMC5651046 29089954

[B132] Seneviratne S. I. Zhang X. Adnan M. Badi W. Dereczynski C. Luca Di A.. (2021). Weather and climate extreme events in a changing climate. In Climate Change 2021: The Physical Science Basis. Contribution of Working Group I to the Sixth Assessment Report of the Intergovernmental Panel Masson-Delmotte V. Zhai P. Pirani A. Connors S.L. Péan C. Berger S. (eds.), (Cambridge, United Kingdom and New York, NY, USA: Cambridge University Press) 1513–1766. doi: 10.1017/9781009157896.013

[B133] Setia R. C. Setia N. (2008). “THE ‘-OMICS’ TECHNOLOGIES AND CROP IMPROVEMENT,” in Crop improvement: strategies and applications, 1–18.

[B134] Sharma L. Priya M. Kaushal N. Bhandhari K. Chaudhary S. Dhankher O. P.. (2020). Plant growth-regulating molecules as thermoprotectants: functional relevance and prospects for improving heat tolerance in food crops. J. Exp. Bot. 71, 569–594. doi: 10.1093/jxb/erz333 31328236

[B135] Singh A. K. Ganapathysubramanian B. Sarkar S. Singh A. (2018). Deep learning for plant stress phenotyping: trends and future perspectives. Trends Plant Sci. 23, 883–898. doi: 10.1016/j.tplants.2018.07.004 30104148

[B136] Sloat L. L. Davis S. J. Gerber J. S. Moore F. C. Ray D. K. West P. C.. (2020). Climate adaptation by crop migration. Nat. Commun. 11, 1243. doi: 10.1038/s41467-020-15076-4 32144261 PMC7060181

[B137] Smýkal P. Nelson M. N. Berger J. D. Von Wettberg E. J. B. (2018). The impact of genetic changes during crop domestication. Agronomy 8, 119. doi: 10.3390/agronomy8070119

[B138] Snowdon R. J. Wittkop B. Chen T.-W. Stahl A. (2021). Crop adaptation to climate change as a consequence of long-term breeding. Theor. Appl. Genet. 134, 1613–1623. doi: 10.1007/s00122-020-03729-3 33221941 PMC8205907

[B139] Soda N. Wallace S. A. Karan R. (2015). Omics study for abiotic stress responses in plants. Plants Agric Res. 2 (1), 28–34. doi: 10.15406/apar.2015.02.00037

[B140] Stella T. Webber H. Olesen J. E. Ruane A. C. Fronzek S. Bregaglio S.. (2021). Methodology to assess the changing risk of yield failure due to heat and drought stress under climate change. Environ. Res. Lett. 16, 104033. doi: 10.1088/1748-9326/ac2196

[B141] Sun Q. Miao C. Hanel M. Borthwick A. G. L. Duan Q. Ji D.. (2019). Global heat stress on health, wildfires, and agricultural crops under different levels of climate warming. Environ. Int. 128, 125–136. doi: 10.1016/j.envint.2019.04.025 31048130

[B142] Sun Q. Zhao Y. Zhang Y. Chen S. Ying Q. Lv Z.. (2022). Heat stress may cause a significant reduction of rice yield in China under future climate scenarios. Sci. Total Environ. 818, 151746. doi: 10.1016/j.scitotenv.2021.151746 34801492

[B143] Takahashi M. Uematsu Y. Kashiwaba K. Yagasaki K. Hajika M. Matsunaga R.. (2003). Accumulation of high levels of free amino acids in soybean seeds through integration of mutations conferring seed protein deficiency. Planta 217, 577–586. doi: 10.1007/s00425-003-1026-3 12684787

[B144] Tardieu F. Cabrera-Bosquet L. Pridmore T. Bennett M. (2017). Plant phenomics, from sensors to knowledge. Curr. Biol. 27, R770–R783. doi: 10.1016/j.cub.2017.05.055 28787611

[B145] Tavan M. Wee B. Brodie G. Fuentes S. Pang A. Gupta D. (2021). Optimizing sensor-based irrigation management in a soilless vertical farm for growing microgreens. Front. Sustain. Food Syst. 4. doi: 10.3389/fsufs.2020.622720

[B146] Templer S. E. Ammon A. Pscheidt D. Ciobotea O. Schuy C. McCollum C.. (2017). Metabolite profiling of barley flag leaves under drought and combined heat and drought stress reveals metabolic QTLs for metabolites associated with antioxidant defense. J. Exp. Bot. 68, 1697–1713. doi: 10.1093/jxb/erx038 28338908 PMC5441916

[B147] Thomason K. Babar M. A. Erickson J. E. Mulvaney M. Beecher C. MacDonald G. (2018). Comparative physiological and metabolomics analysis of wheat (Triticum aestivum L.) following post-anthesis heat stress. PloS One 13, e0197919. doi: 10.1371/journal.pone.0197919 29897945 PMC5999278

[B148] Tigchelaar M. Battisti D. S. Naylor R. L. Ray D. K. (2018). Future warming increases probability of globally synchronized maize production shocks. Proc. Natl. Acad. Sci. 115, 6644–6649. doi: 10.1073/pnas.1718031115 29891651 PMC6042138

[B149] Timsina J. (2018). Can organic sources of nutrients increase crop yields to meet global food demand? Agronomy 8, 214. doi: 10.3390/agronomy8100214

[B150] Toriyama K. (2020). Development of precision agriculture and ICT application thereof to manage spatial variability of crop growth. Soil Sci. Plant Nutr. 66, 811–819. doi: 10.1080/00380768.2020.1791675

[B151] Ul Hassan M. Rasool T. Iqbal C. Arshad A. Abrar M. Abrar M. M.. (2021). Linking plants functioning to adaptive responses under heat stress conditions: A mechanistic review. J. Plant Growth Regul 41, 2596–2613. doi: 10.1007/s00344-021-10493-1

[B152] Varshney R. K. Terauchi R. McCouch S. R. (2014). Harvesting the promising fruits of genomics: applying genome sequencing technologies to crop breeding. PloS Biol. 12, e1001883. doi: 10.1371/journal.pbio.1001883 24914810 PMC4051599

[B153] Venios X. Korkas E. Nisiotou A. Banilas G. (2020). Grapevine responses to heat stress and global warming. Plants 9, 1754. doi: 10.3390/plants9121754 33322341 PMC7763569

[B154] Vu L. D. Zhu T. Verstraeten I. van de Cotte B. Gevaert K. De Smet I. (2018). Temperature-induced changes in the wheat phosphoproteome reveal temperature-regulated interconversion of phosphoforms. J. Exp. Bot. 69, 4609–4624. doi: 10.1093/jxb/ery204 29939309 PMC6117581

[B155] Wang J. Gan Y. T. Clarke F. McDonald C. L. (2006). Response of chickpea yield to high temperature stress during reproductive development. Crop Sci. 46, 2171–2178. doi: 10.2135/cropsci2006.02.0092

[B156] Wang L. Ma K.-B. Lu Z.-G. Ren S.-X. Jiang H.-R. Cui J.-W.. (2020). Differential physiological, transcriptomic and metabolomic responses of Arabidopsis leaves under prolonged warming and heat shock. BMC Plant Biol. 20, 86. doi: 10.1186/s12870-020-2292-y 32087683 PMC7036190

[B157] Wani S. H. (2019). Recent approaches in omics for plant resilience to climate change (Springer International Publishing). doi: 10.1007/978-3-030-21687-0

[B158] Watt M. Fiorani F. Usadel B. Rascher U. Muller O. Schurr U. (2020). Phenotyping: new windows into the plant for breeders. Annu. Rev. Plant Biol. 71, 689–712. doi: 10.1146/annurev-arplant-042916-041124 32097567

[B159] Xu G. Singh S. K. Reddy V. R. Barnaby J. Y. Sicher R. C. Li T. (2016). Soybean grown under elevated CO 2 benefits more under low temperature than high temperature stress: Varying response of photosynthetic limitations, leaf metabolites, growth, and seed yield. J. Plant Physiol. 205, 20–32. doi: 10.1016/j.jplph.2016.08.003 27589223

[B160] Yadav M. R. Choudhary M. Singh J. Lal M. K. Jha P. K. Udawat P.. (2022). Impacts, tolerance, adaptation, and mitigation of heat stress on wheat under changing climates. Int. J. Mol. Sci. 23, 2838. doi: 10.3390/ijms23052838 35269980 PMC8911405

[B161] Yamakawa H. Hakata M. (2010). Atlas of rice grain filling-related metabolism under high temperature: joint analysis of metabolome and transcriptome demonstrated inhibition of starch accumulation and induction of amino acid accumulation. Plant Cell Physiol. 51, 795–809. doi: 10.1093/pcp/pcq034 20304786 PMC2871029

[B162] Yang H. Gu X. Ding M. Lu W. Lu D. (2018). Heat stress during grain filling affects activities of enzymes involved in grain protein and starch synthesis in waxy maize. Sci. Rep. 8, 15665. doi: 10.1038/s41598-018-33644-z 30353095 PMC6199321

[B163] Zampieri M. Weissteiner C. J. Grizzetti B. Toreti A. van den Berg M. Dentener F. (2020). Estimating resilience of crop production systems: From theory to practice. Sci. Total Environ. 735, 139378. doi: 10.1016/j.scitotenv.2020.139378 32480148 PMC7374405

[B164] Zandalinas S. I. Fritschi F. B. Mittler R. (2021). Global warming, climate change, and environmental pollution: recipe for a multifactorial stress combination disaster. Trends Plant Sci. 26, 588–599. doi: 10.1016/j.tplants.2021.02.011 33745784

[B165] Zandalinas S. I. Mittler R. (2022). Plant responses to multifactorial stress combination. New Phytol. 234, 1161–1167. doi: 10.1111/nph.18087 35278228

[B166] Zandalinas S. I. Sales C. Beltrán J. Gómez-Cadenas A. Arbona V. (2017). Activation of secondary metabolism in citrus plants is associated to sensitivity to combined drought and high temperatures. Front. Plant Sci. 7. doi: 10.3389/fpls.2016.01954 PMC5220112 28119698

[B167] Zenda T. Liu S. Dong A. Li J. Wang Y. Liu X.. (2021). Omics-facilitated crop improvement for climate resilience and superior nutritive value. Front. Plant Sci. 12. doi: 10.3389/fpls.2021.774994 PMC8672198 34925418

[B168] Zhang C. Hiradate S. Kusumoto Y. Morita S. Koyanagi T. F. Chu Q.. (2021). Ionomic responses of local plant species to natural edaphic mineral variations. Front. Plant Sci. 12. doi: 10.3389/fpls.2021.614613 PMC8039527 33854517

[B169] Zhang F. Neik T. X. Thomas W. J. W. Batley J. (2023). CRISPR-based genome editing tools: an accelerator in crop breeding for a changing future. Int. J. Mol. Sci. 24, 8623. doi: 10.3390/ijms24108623 37239967 PMC10218198

[B170] Zhao C. Liu B. Piao S. Wang X. Lobell D. B. Huang Y.. (2017). Temperature increase reduces global yields of major crops in four independent estimates. Proc. Natl. Acad. Sci. U. S. A. 114, 9326–9331. doi: 10.1073/pnas.1701762114 28811375 PMC5584412

[B171] Zhao J. Lu Z. Wang L. Jin B. (2020). Plant responses to heat stress: physiology, transcription, noncoding RNAs, and epigenetics. Int. J. Mol. Sci. 22, 117. doi: 10.3390/ijms22010117 33374376 PMC7795586

[B172] Zhou R. Jiang F. Niu L. Song X. Yu L. Yang Y.. (2022). Increase crop resilience to heat stress using omic strategies. Front. Plant Sci. 13. doi: 10.3389/fpls.2022.891861 PMC9152541 35656008

[B173] Zhou Y. Xu F. Shao Y. He J. (2022). Regulatory mechanisms of heat stress response and thermomorphogenesis in plants. Plants 11, 3410. doi: 10.3390/plants11243410 36559522 PMC9788449

[B174] Zhu P. Burney J. Chang J. Jin Z. Mueller N. D. Xin Q.. (2022). Warming reduces global agricultural production by decreasing cropping frequency and yields. Nat. Clim. Change 12, 1016–1023. doi: 10.1038/s41558-022-01492-5

[B175] Zulfiqar F. Navarro M. Ashraf M. Akram N. A. Munné-Bosch S. (2019). Nanofertilizer use for sustainable agriculture: Advantages and limitations. Plant Sci. 289, 110270. doi: 10.1016/j.plantsci.2019.110270 31623775

